# From Synthesis to Scrutiny: Evaluating the Quality of Anaesthesiology Meta-Analyses (2015-2022)

**DOI:** 10.7759/cureus.90058

**Published:** 2025-08-14

**Authors:** Jeffrey Turner, Jai-kieron Patel, Kirsty Fraser

**Affiliations:** 1 Anaesthesiology, Queen Elizabeth Hospital Birmingham, Birmingham, GBR; 2 Anaesthesiology, The York Hospital, York, GBR; 3 Anaesthesiology, University Hospital of North Durham, Durham, GBR

**Keywords:** anaesthesiology, anaesthetic research, anaesthetics, meta-research, pain, systematic review and meta-analysis

## Abstract

Introduction

Meta-analyses (MAs) are useful for informing evidence-based clinical practice in constantly evolving fields such as anaesthesiology. This study aimed to assess the methodological quality of meta-analyses published in leading anaesthesiology journals from 2015 to 2022 and to evaluate trends in quality over time.

Methods

We utilised the Revised-A MeaSurement Tool to Assess systematic Reviews (R-AMSTAR) tool with in-depth binary sub-criterion scoring to evaluate the methodological rigour of a selection of meta-analyses identified in six prominent anaesthesiology journals between 2015 and 2022. For the purpose of this study, the journals selected for review were as follows: Anaesthesia, British Journal of Anaesthesia, Anesthesia & Analgesia, Anesthesiology, BMC Anesthesiology and Pain. PubMed and individual journals were manually searched for meta-analyses published within this timeframe, and a sample of papers was selected for full-text review and appraisal against the R-AMSTAR criteria. Areas on the R-AMSTAR tool that scored poorly were highlighted, and comparisons were made across journals and over time.

Results

Our study identified 455 meta-analyses published in leading anaesthesiology journals during the study period and found an increase in the number of meta-analyses published over time. In-depth R-AMSTAR analysis of 130 randomly selected papers found that, despite this increase, the overall methodological quality of these meta-analyses did not significantly improve over time, indicating a plateau in quality post-2014, in contrast to earlier reported trends. No statistically significant differences in methodological quality or sub-criterion level adherence were observed between the included journals. Consistent strengths were identified in reporting duplicate data extraction and descriptions of included studies. Conversely, persistent weaknesses were noted in research planning, the explicit statistical assessment of publication bias and the use of study quality to guide conclusions, as well as transparent reporting of excluded studies.

Conclusion

While the methodological quality of meta-analyses in anaesthesiology remained stable between 2015 and 2022, the anticipated trajectory of continued improvement has not materialised in recent years. Core methodological criteria, particularly regarding publication bias assessment, utilising study quality to inform conclusions and transparency in reporting excluded studies, continue to be under-implemented. Future efforts should focus on renewed editorial emphasis and targeted author training to enhance transparency and rigour in reporting practices to ensure the highest standards of scientific reliability.

## Introduction

Meta-analyses (MAs) serve as a useful instrument in the field of anaesthesiology, providing a quantitative synthesis of existing research to inform clinical practice and improve patient care. These studies combine the results of multiple primary studies, offering a higher level of evidence compared to individual trials [1]. However, the validity and reliability of meta-analyses are intrinsically linked to their methodological rigour, thereby necessitating a thorough evaluation of their quality [2].

The A MeaSurement Tool to Assess systematic Reviews (AMSTAR) tool is a widely recognised and validated instrument designed to evaluate the methodological quality of systematic reviews and meta-analyses [3]. This tool enables a structured and thorough evaluation, including key elements such as the comprehensiveness and reproducibility of the search strategy, the objectivity and appropriateness of study selection criteria, the accuracy and consistency of data extraction procedures, the validity of the risk of bias assessments and the appropriateness and robustness of statistical analyses employed.

The Revised-AMSTAR (R-AMSTAR) tool was developed as a more rigorous tool applicable to overcome limitations associated with its predecessor and provide a more comprehensive assessment of systematic review quality [4]. The R-AMSTAR tool has 11 items for appraising the methodological quality of systematic reviews, with each item assigned a score from 1 to 4. A high-quality meta-analysis, as determined by R-AMSTAR, demonstrates meticulous adherence to established standards, minimising the risk of bias and ensuring reliable and valid conclusions that can confidently inform clinical decision-making. Conversely, a meta-analysis deemed to be of critically low quality exhibits substantial methodological flaws that may compromise the integrity of its findings, thereby limiting its utility in guiding clinical practice. Despite an increasing awareness of the importance of methodological rigour, many published meta-analyses still exhibit methodological deficiencies [5]. In addition, the number of systematic reviews published in anaesthetic journals has consistently increased in recent years [6]. Despite increased output, it remains important that systematic reviews and meta-analyses are always conducted to the highest possible standard and that they are accurately and transparently reported. It is therefore essential to assess the quality of meta-analyses to ensure that clinicians are using the best available evidence to make decisions about patient care.

The objectives of this study are to identify all meta-analyses published in six prominent anaesthesiology journals between 2015 and 2022, evaluate the methodological quality of these meta-analyses using the R-AMSTAR tool and assess trends in the methodological quality of meta-analyses in anaesthesiology over time. The results of this study will provide insights into the current state of meta-analysis quality in anaesthesiology and highlight areas for improvement.

## Materials and methods

Study selection

This study included meta-analyses published in six prominent anaesthesiology journals between January 1, 2015, and December 31, 2022. For the purpose of this study, the journals selected for review were as follows: Anaesthesia, British Journal of Anaesthesia, Anesthesia & Analgesia, Anesthesiology, BMC Anesthesiology and Pain.

Studies were included if they met the following criteria: The study was a meta-analysis defined as a quantitative synthesis of data from multiple primary studies, the study was initially published in one of the six anaesthesiology journals as detailed and the study must be published between January 1, 2015, and December 31, 2022.

The following types of studies were excluded: all Cochrane reviews, as these are allowed to be cross-published and are already quality appraised using a formal process. Also excluded were narrative reviews, systematic reviews without a meta-analysis, meta-analyses of non-clinical studies, meta-analyses not related to anaesthesiology, retractions of published meta-analysis studies and duplicate publications. Furthermore, meta-analyses of individual patient data were excluded because they do not incorporate traditional meta-analytical techniques.

Search strategy and data extraction

A comprehensive search of the six anaesthesiology journals was conducted to identify potentially eligible meta-analyses. The search involved manual screening of each journal issue published between January 1, 2015, and December 31, 2022. In addition, we searched within each journal's website using the terms 'meta-analysis' and 'Systematic review' to identify potentially missed articles. Two reviewers independently screened the titles and abstracts of the identified articles for eligibility, and at any point of uncertainty, the full-text article was reviewed. Disagreements between the two reviewers were resolved by discussion and the involvement of a third independent reviewer. A random selection of 25% of the articles was selected for data extraction and quality assessment. The sample size was selected to provide satisfactory power to statistical tests while ensuring that all papers could be reviewed in a reasonable timeframe. The publishing journal and date of the randomly selected papers were checked and verified to ensure that the sample of papers selected was representative of the total body of meta-analyses published in the six selected journals during the period of study. In the case of this study, six additional papers were randomly selected from a stratified sample of papers to top up under-represented years and journals. Two authors assessed each of the randomly selected papers against each of the 44 points on the R-AMSTAR checklist.

Data analysis

The overall R-AMSTAR score was calculated for each meta-analysis, and the distribution of quality ratings was calculated by publishing date, and the journal was assessed using Pearson's correlation. Trends in R-AMSTAR scores over time were analysed using linear regression to assess whether the methodological quality of meta-analyses has improved, declined or remained stable during the study period. Chi-squared tests of independence were performed for all R-AMSTAR sub-criteria to assess whether adherence differed between journals, and a Kruskal-Wallis H test was performed to assess whether R-AMSTAR scores differed significantly between journals. In addition, specific points of the R-AMSTAR checklist were highlighted where published meta-analyses consistently perform well or poorly.

## Results

**Figure 1:**
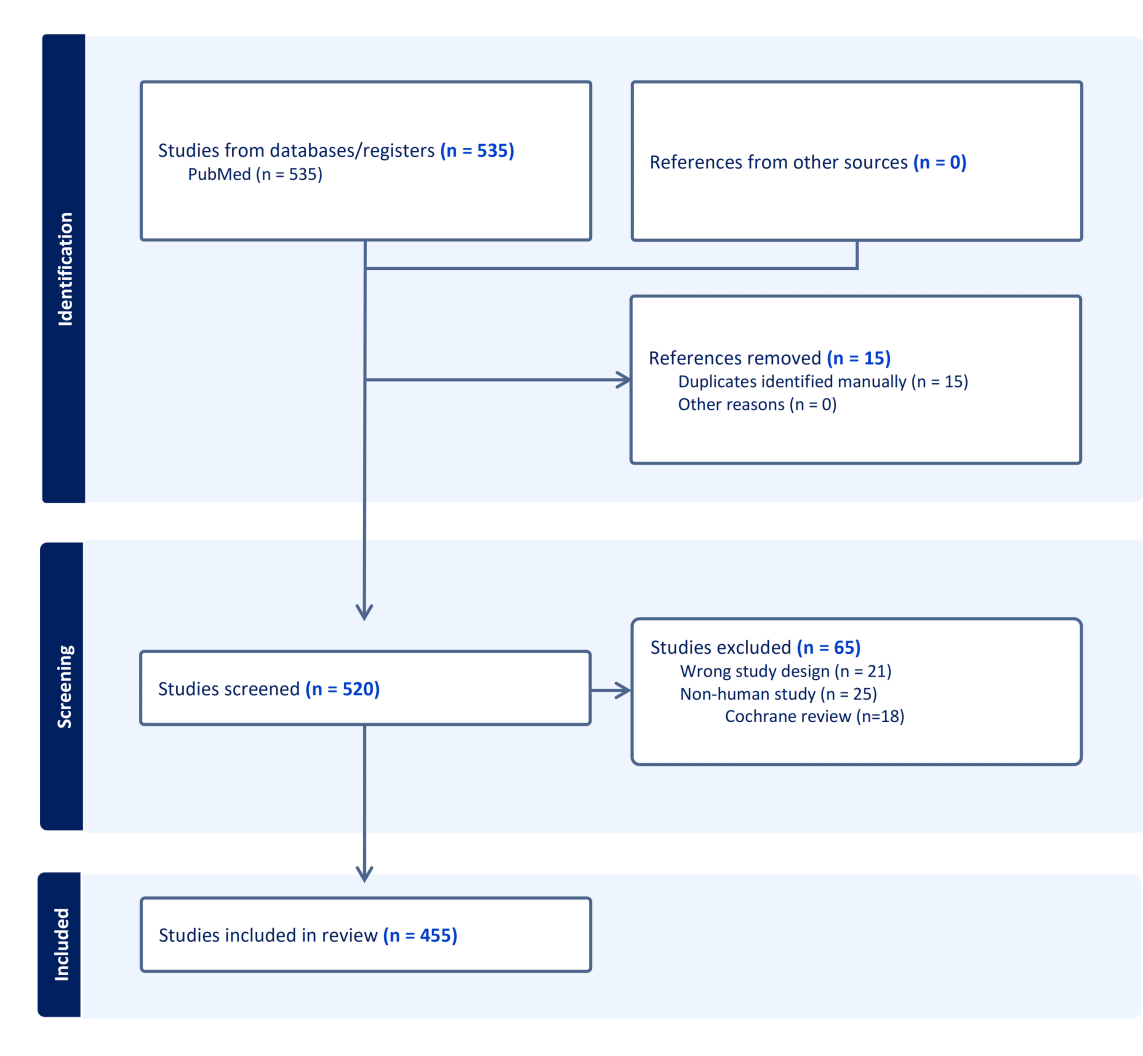
PRISMA 2020 Flow Diagram PRISMA: Preferred Reporting Items for Systematic Reviews and Meta-Analyses

The initial search yielded 535 articles. After screening titles, abstracts and full article texts when required, 455 papers satisfied the inclusion and exclusion criteria. From the pool of eligible articles, 114 papers (25%) were randomly selected for in-depth analysis; an additional 16 papers were randomly selected from journals and years under-represented in the initial random sample. A total of 130 papers were assessed using the R-AMSTAR criteria. Figure 1 details the study selection process in the form of a Preferred Reporting Items for Systematic Reviews and Meta-Analyses (PRISMA) flowchart. A full list of the included studies is available at the following: https://doi.org/10.6084/m9.figshare.29755505.v1.

**Figure 2:**
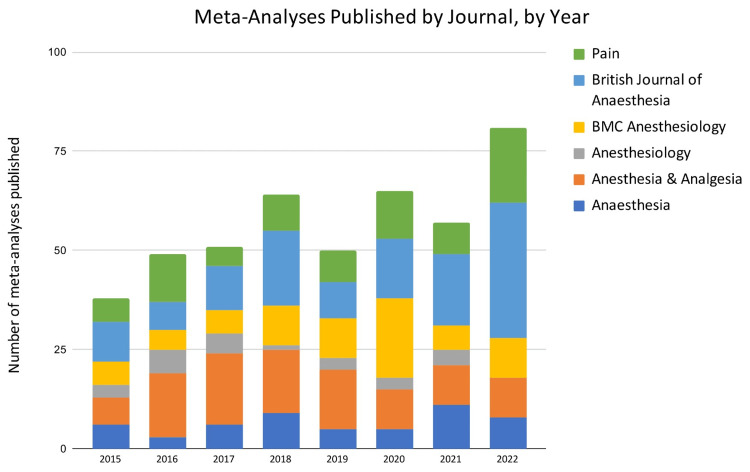
Bar Chart Showing the Number of Meta-Analyses Published by Journal and by Year

A review of meta-analysis publications in six anaesthesiology journals from 2015 to 2022 revealed variations in the number of published papers per journal and year, as detailed in Figure 2. The British Journal of Anaesthesia exhibited the highest overall output, with a notable increase in publications in 2022. Anesthesia & Analgesia consistently published a substantial number of MAs throughout the period, while Anesthesiology had the lowest output among the selected journals. Anaesthesia and Pain demonstrated moderate and fluctuating publication rates over the years. Total meta-analysis publications increased from 2015 to 2022, with Pearson's correlation (r) of 0.822 (p = 0.012) suggesting a strong positive overall trend.

**Figure 3:**
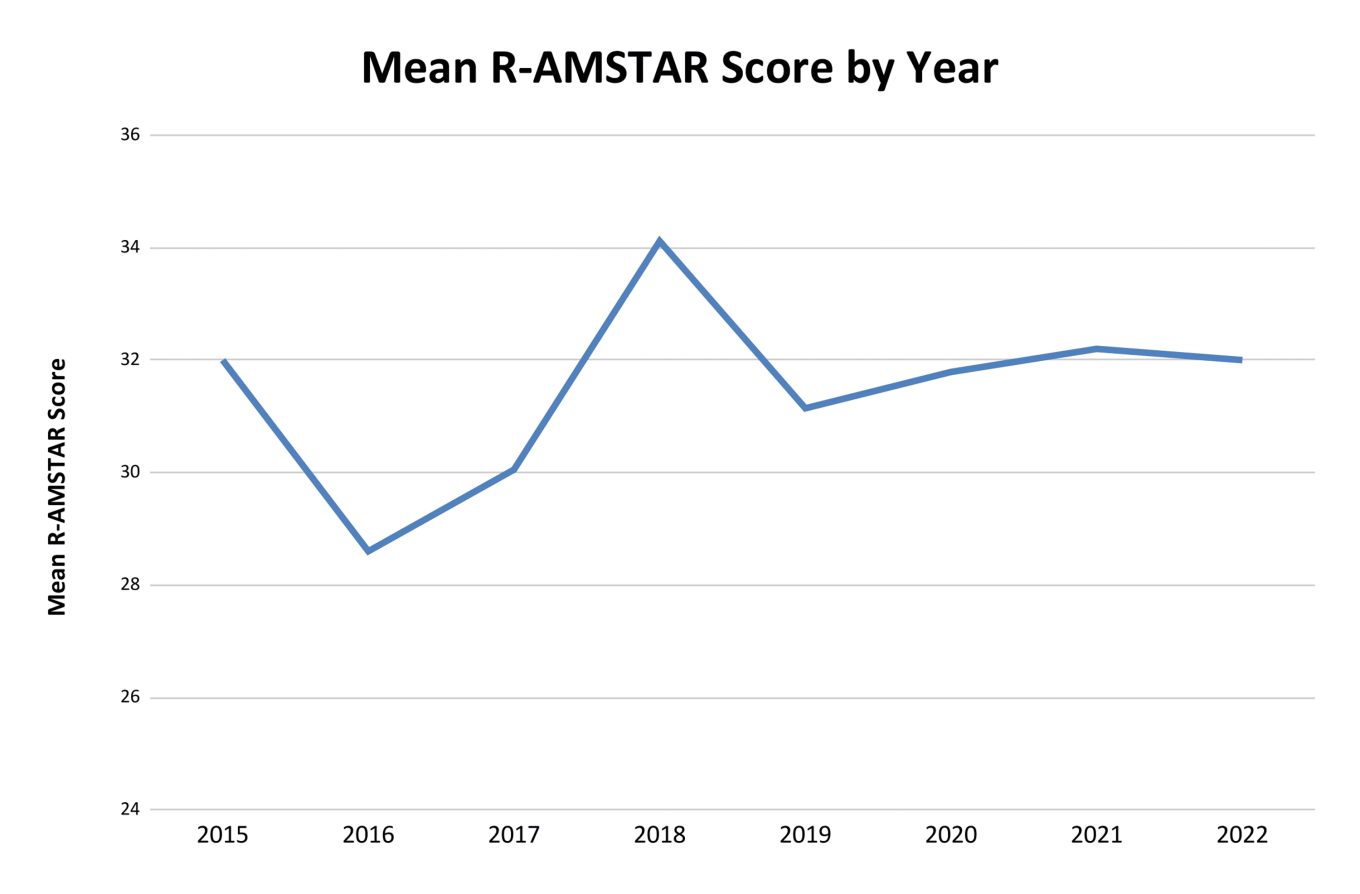
Line Graph Showing the Mean R-AMSTAR Score by all Examined Journals and by Year R-AMSTAR: Revised-A MeaSurement Tool to Assess systematic Reviews

The mean R-AMSTAR score across the sample was 31.63 (standard deviation {SD}: 4.65; 95% CI: 30.82-32.44), with scores ranging from 11 to 41 out of a maximum possible score of 44. The median score was 33 (IQR: 30-34), indicating generally moderate to high methodological quality. Temporal analysis showed no statistically significant trend in methodological quality over time (linear regression β = 0.186; p = 0.298). Although minor fluctuations in yearly averages were observed, no clear trend was evident between 2015 and 2022, as seen in Figure 3.

**Figure 4:**
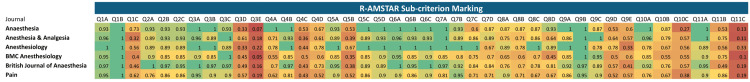
Heat Map Showing the Mean Sub-criterion R-AMSTAR Score of Each Examined Journal R-AMSTAR: Revised-A MeaSurement Tool to Assess systematic Reviews

The analysis of individual sub-criteria revealed high adherence to items related to duplicate data extraction, a priori design and comprehensive literature search strategies. Conversely, criteria such as reporting excluded studies, assessing publication bias using statistical methods and incorporating quality assessments into conclusions were infrequently met. To explore whether adherence to individual sub-criteria varied by journal, a heatmap was generated based on the average binary score per sub-criterion across journals (Figure 4). Additionally, a chi-squared test of independence was performed across all binary sub-criterion values. The result was not statistically significant (χ² = 44.22; p = 1.00), indicating that sub-criterion-level adherence does not meaningfully differ between journals.

**Figure 5:**
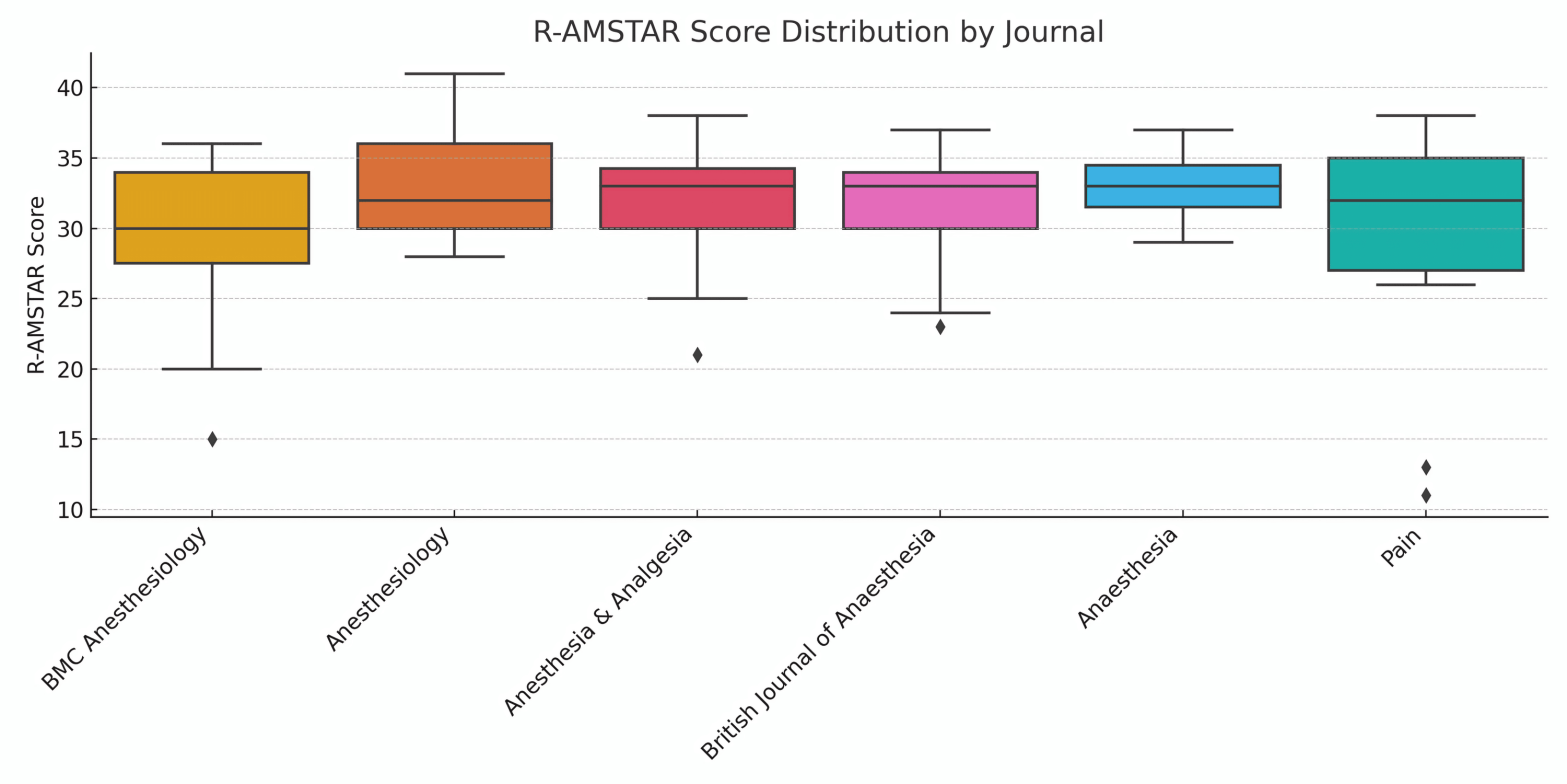
A Box Plot of the R-AMSTAR Scores for Each Examined Journal R-AMSTAR: Revised-A MeaSurement Tool to Assess systematic Reviews

A Kruskal-Wallis H test assessed whether R-AMSTAR scores differed significantly between journals with fewer than five included studies. The result was not statistically significant (H = 4.53; p = 0.476), suggesting no substantial difference in methodological quality among journals. Mean scores and standard deviations for each journal are depicted in Figure 5.

## Discussion

This study assessed the methodological quality of meta-analyses published in prominent anaesthesiology journals from 2015 to 2022 using the R-AMSTAR tool. Our analysis of anaesthetic reviews published over time is consistent with Zou et al.'s findings that the number of meta-analyses published over time in the field of anaesthetics is increasing [6]. Despite a general increase in the number of meta-analyses published in anaesthesiology journals, the overall methodological quality has not significantly improved over time.

Our results do not corroborate those of hand surgery and spine surgery reviews, where a significant increase in median AMSTAR score was observed over time [7,8]. There are several possible explanations for this result. First, it is plausible that the field of anaesthesiology has already reached a relatively high baseline level of methodological rigour in its meta-analytical practices, leaving less room for substantial improvement compared to other specialties. This is supported by the findings from similar studies in other research areas, for example, in heart failure, where a significant proportion of meta-analyses were rated as critically low quality [9]. Second, the specific journals included in our analysis already enforce stringent methodological standards, where easier-to-achieve criteria have been met at baseline for submission, leading to a point of diminishing returns where further improvements are difficult to achieve. Alternatively, the limited duration of our study, spanning from 2015 to 2022, may not be sufficiently extensive to capture more subtle yet significant shifts in methodological practices.

However, when focussing on anaesthetics alone and comparing results with Hall et al., who reviewed meta-analyses published in anaesthetic journals between 2005 and 2014, our results offer a more recent update and suggest that improvements in methodological quality may have plateaued [10]. Hall et al. reported a positive temporal trend (rs = 0.24; p < 0.001), while our analysis found no statistically significant change in scores over the subsequent eight-year period [10]. The difference may be partly attributable to our use of binary sub-criterion scoring rather than ordinal ratings, potentially offering a stricter assessment of methodological completeness. Alternatively, the plateau may reflect a point of diminishing returns in methodological education and refinement, given that the methodological quality of the reviewed papers was already generally high.

The summary scores for individual R-AMSTAR questions provide further insight into specific strengths and weaknesses across the field. High scores in Q2 and Q6 suggest that duplicate data extraction and description of included studies are well-reported practices. In contrast, Q1 (a priori design), Q7 (the assessment of study quality) and Q8 (the use of quality in formulating conclusions) show consistently low means, highlighting ongoing challenges in research planning and interpretation.

The methodological quality of the included primary studies is critical to the validity and reliability of a meta-analysis. Low scores in Q7 suggest that authors are not sufficiently critically appraising the primary papers included in the meta-analysis. Without the careful selection of high-quality evidence, meta-analyses risk synthesising flawed or biased research, leading to potentially misleading conclusions. There are cases where authors must include lower-quality papers, in particular in emergent fields where few high-quality trials have been performed. However, low scores in Q8 suggest that authors are not considering the quality of the included studies when interpreting the results of the meta-analysis.

Comparison between journals revealed no statistically significant differences in mean methodological quality scores, supporting earlier findings that suggest consistency in quality among leading anaesthesiology journals. The visualisation in Figure 4 further reinforces the similar distribution of scores, though some variation in spread and outliers may reflect differing editorial emphasis or author practices. This suggests that methodological practices are largely consistent across publications, and areas of strength or weakness are shared rather than journal-specific.

Similar to Hall et al., our study identified persistent weaknesses in several R-AMSTAR domains [10]. Notably, the inclusion of lists of excluded studies, explicit statistical assessments of publication bias (e.g. Egger's test) and the use of study quality to guide conclusions remain underreported. These deficits represent important targets for methodological training and journal policy.

The strengths of this study include its recent sample, granular criterion-level scoring and alignment with validated R-AMSTAR methodology. However, limitations include the use of binary scoring, which does not account for partial adherence or quality gradation, and the exclusion of meta-analyses in journals outside the commonly indexed anaesthesiology publications. This paper focussed on the prominent anaesthesiology journals; however, further studies into less prominent, open-access and pay-to-publish journals may reveal different and informative patterns.

In conclusion, while the methodological quality of anaesthesiology meta-analyses remains reasonably strong, several core criteria continue to be under-implemented. The lack of continued improvement post-2014 may indicate a need for renewed editorial emphasis and author training. Future efforts should focus on enhancing transparency and rigour in reporting practices, particularly for criteria known to influence validity and reproducibility. In addition, journals could include an AMSTAR assessment as a part of their evaluation of publishability.

## Conclusions

Meta-analyses serve a significant role in evidence-based clinical practice, particularly in rapidly evolving fields such as anaesthesiology, where their findings inform guidelines and policy. This updated evaluation reveals that while the overall methodological quality of these reviews remains steady, the anticipated trajectory of continued improvement has not materialised in recent years. Persistent shortfalls in key domains such as publication bias assessment, the use of study quality to inform conclusions and reporting transparency of excluded studies are areas where authors, reviewers and editors should collectively focus their efforts. Targeted educational initiatives, coupled with clearer editorial requirements (including AMSTAR appraisal), may re-energise progress and ensure that meta-analyses meet the highest standards of scientific rigour and reliability.
